# Regulation of Sema3c and the Interaction between Cardiac Neural Crest and Second Heart Field during Outflow Tract Development

**DOI:** 10.1038/s41598-017-06964-9

**Published:** 2017-07-28

**Authors:** Kazuki Kodo, Shinsuke Shibata, Sachiko Miyagawa-Tomita, Sang-Ging Ong, Hiroshi Takahashi, Tsutomu Kume, Hideyuki Okano, Rumiko Matsuoka, Hiroyuki Yamagishi

**Affiliations:** 1 Department of Pediatrics, Division of Pediatric Cardiology, Keio University School of Medicine, Tokyo, 160-8582 Japan; 2 Department of Physiology, Keio University School of Medicine, Tokyo, 160-8582 Japan; 3 Department of Pediatric Cardiology, Tokyo Women’s Medical University, Tokyo, 162-8666 Japan; 4 Stanford Cardiovascular Institute, Stanford University School of Medicine, Stanford, CA 94305-5111 USA; 5 Department of Neurology, National Hospital Organization, Tottori Medical Center, Tottori Tottori, 689-0203 Japan; 6 Feinberg Cardiovascular Research Institute, Feinberg School of Medicine, Northwestern University, Chicago, IL 60611 USA

## Abstract

The cardiac neural crest cells (cNCCs) and the second heart field (SHF) play key roles in development of the cardiac outflow tract (OFT) for establishment of completely separated pulmonary and systemic circulations in vertebrates. A neurovascular guiding factor, Semaphorin 3c (Sema3c), is required for the development of the OFT, however, its regulation of the interaction between cNCCs and SHF remains to be determined. Here, we show that a Sema3c is a candidate that mediates interaction between cNCCs and the SHF during development of the OFT. Foxc1/c2 directly activates the transcription of Sema3c in the OFT, whereas, a hypomorph of Tbx1, a key SHF transcription factor, resulted in the ectopic expression of Sema3c in the pharyngeal arch region. Fgf8, a downstream secreted factor of Tbx1, inhibited the expression of Sema3c in cNCCs via activation of ERK1/2 signaling. Blocking of FGF8 caused ectopic expression of SEMA3C and a migration defect of cNCCs, resulting in abnormal chick pharyngeal arch development. These results suggest that proper spatio-temporal expression of Sema3c, regulated positively by Foxc1/c2 and negatively by the Tbx1-Fgf8 cascade, respectively, is essential for the interaction between cNCCs and the SHF that correctly navigates cNCCs towards the OFT, composed of SHF-derived cells.

## Introduction

The neural crest is a multipotent and transient migratory lineage that gives rise to various different cell types^1^. Neural crest cells present in the region from the mid-otic placode to the caudal limit of somite 3 are referred to as cardiac neural crest cells (cNCCs)^2^. cNCCs migrate ventrally to form the third, fourth, and sixth pharyngeal arch arteries. They migrate further into the cardiac outflow tract (OFT) to give rise to the septum which divides a single great vessel arising from the embryonic heart, the truncus arteriosus, into the aorta and pulmonary trunk^3^.

Cardiac progenitor cells from a second source, termed the second heart field (SHF), are required in addition to cNCCs for formation of the OFT. Progenitor cells derived from the SHF in the pharyngeal mesoderm migrate into the embryonic heart tube from both the arterial and venous poles where they subsequently construct the OFT and atria, respectively^4–6^. Numerous transcription factors, including Isl1, Foxc1, Foxc2, and Tbx1, and signaling molecules including Fgfs, Bmps, and Wnts, have been reported to play a role in development of the SHF^7^.

In a recent study, it was found that Wnt1-positive neural crest cells form part of the intrapericardial aortic trunk, whereas lineages positive for the SHF-marker transcription factor Six2 give rise to the intrapericardial pulmonary trunk. This suggests that the aortic and pulmonary trunks are derived from cNCCs and SHF progenitors, respectively^8^. Because cNCCs and the SHF are essential for proper development of the OFT^9^, understanding the mechanisms that regulate the development of cNCCs and their interactions with the SHF will allow these findings to be applied in clinical practice.

Class 3 semaphorins are secreted proteins that act as axon repellents or attractants, controlling the formation of neuronal connections. They are also expressed in non-neuronal tissues and regulate cardiac morphogenesis and angiogenesis^10^. Semaphorin 3c (Sema3c) is a neurovascular guiding molecule of the class 3 semaphorin family that is implicated in cardiac development. Mice lacking Sema3c exhibit interruption of the aortic arch and persistent truncus arteriosus; thus Sema3c is thought to regulate the development and migration of cNCCs^11^. During the early stages of heart development, *Sema3c* is expressed in the OFT as well as in the pharyngeal arch region that contains cardiac progenitor niches composed of SHF progenitor cells and cNCCs^12^. Previous studies showed that a transcription factor, Gata6, regulates the expression of Sema3c and its receptor plexin A2, during development of the OFT^13, 14^. Taken together, Sema3c signaling from the OFT may be essential for migration of cNCCs towards the OFT; however, regulation of Sema3c expression is yet to be elucidated.

Here, we show that Foxc1/Foxc2 and Tbx1-Fgf8 signaling are essential positive and negative regulators of Sema3c expression, respectively, and contribute to the development and migration of cNCCs for OFT formation during embryogenesis. Our results suggest that Sema3c is a key signaling molecule that mediates interaction between cNCCs and the SHF, which is implicated in proper septation of the OFT and is critical to establishing separate systemic and pulmonary circulation systems.

## Results

### Foxc1/c2 is necessary for the expression of Sema3c in the OFT region

**Figure 1:**
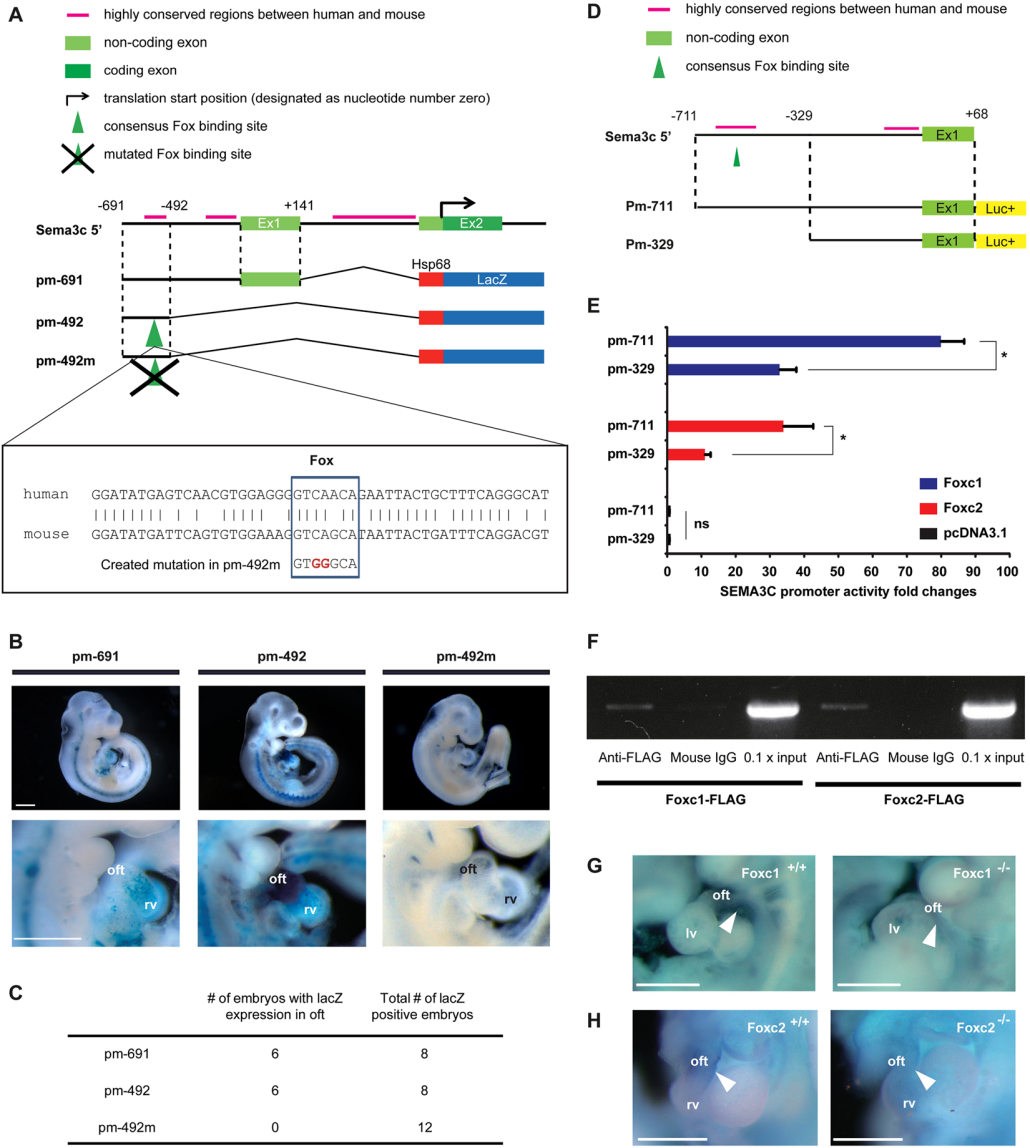
*Sema3c* expression in the cardiac outflow tract (oft) is directly controlled by Foxc1 and Foxc2. (**A**) Genomic organization of the 5′ mouse *Sema3c* locus and flanking region. Green Boxes indicate exons (Ex), black bars indicate highly conserved regions between human and mouse, and translation start site (arrow) is designated as nucleotide number zero. Each construct number is indicated on the left. The consensus Fox binding site is indicated by a green arrowhead. The construct pm-492m has a mutated Fox site in the context of pm-492. Conserved alignments of genomic sequences including the Fox site (box) between human (upper) and mouse (lower) are shown. (**B**) Right lateral views of the hearts of representative embryos obtained with each construct. Lower lane pictures show higher magnification view of the heart. (**C**) The summary of the number of F0 transgenic embryos with specific lacZ expression in the oft and the total number of F0 embryos with lacZ positivity. (**D**) Luciferase reporter constructs under control of the two *Sema3c* genomic DNA fragments (pm-711 and pm-329). (**E**) Relative luciferase activities after co-transfection of reporter constructs with Foxc1 (blue) or Foxc2 (red) expression constructs, or empty vector (pcDNA3.1, black). (**F**) ChIP analysis reveals direct binding of Foxc1 and Foxc2 to the *Sema3c* 5′ sequence (region from −691 to −492) including the Fox site. (**G,H**) DIG-labeled whole mount *in situ* hybridization of mouse embryos at E10.5 showed that *Sema3c* was expressed, but partially down-regulated in the oft of *Foxc1* or *Foxc2* mutant ((**G**) *Foxc1* ^−/−^; (**H**) *Foxc2* ^−/−^) embryos compared with wild-type (**G**, *Foxc1* ^+/+^; **H**, *Foxc2* ^+/+^) embryos. rv, right ventricle; lv, left ventricle; oft, outflow tract. Scale bars, 500 μm. *p < 0.05. Error bars represent s.e.m.

In order to delineate the molecular mechanisms that regulate Sema3c expression during cardiac development, we analyzed *Sema3c* genomic sequences and searched for *cis*-regulatory elements that control *Sema3c* expression. Our transgenic approaches revealed that a 0.7 kb genomic sequence in the *Sema3c* 5′-flanking region, which includes exon 1, could activate Sema3c expression in the OFT and pharyngeal arches at embryonic day (E) 10.5 (Fig. 1A–C: pm-691).

To localize the regulatory elements responsible for Sema3c expression in the embryonic OFT and pharyngeal arches, we constructed multiple genomic fragments of the *Sema3c* 5′ region and tested their ability to direct lacZ expression under a heterologous promoter (*hsp68*)^15^. F0 transgenic mouse embryos harboring a 0.2 kb transgene from −691 to −492 of the *Sema3c* 5′ region (pm-492) showed X-gal staining in the OFT and pharyngeal arches at E10.5, reminiscent of transgenic mouse embryos with pm-691 (Fig. 1A–C: pm-492). Comparison of this 0.2 kb DNA sequence between mice and humans revealed a highly conserved consensus binding site for the winged helix/forkhead (Fox) transcription factors (Fig. 1A). A mutation in this Fox binding site (pm-492m) abolished lacZ expression in the OFT and pharyngeal arches (Fig. 1A–C: pm-492m), suggesting that this Fox binding site is essential for *Sema3c* expression in the embryonic OFT.

To test whether Fox transcription factors could directly activate Sema3c expression through the identified Fox binding site, we performed luciferase assays using co-expression of Foxc1, Foxc2 and numerous transcription factors that are essential for heart development with the reporter construct including the localized Sema3c contiguous sequences (pm-711: from −711 to +68) (Fig. 1D). Our analyses showed that Foxc1 and Foxc2 could activate the pm-711 luciferase significantly at much higher levels than any of the other tested transcription factors could. (Fig. 1E and Supplementary Figure S1). Deletion of the regulatory element, including the Fox binding site (pm-329), resulted in significantly decreased transcriptional activity of the luciferase reporter by Foxc1 and Foxc2 (Fig. 1E). ChIP assay demonstrated that both Foxc1 and Foxc2 could directly bind to the DNA fragment including the identified Fox binding site (Fig. 1F). Next, we analyzed the *in vivo* expression pattern of Sema3c mRNA. At E10.5, Sema3c mRNA expression in the OFT was significantly decreased in Foxc1 or Foxc2 null embryos as compared to wild-type embryos (Fig. 1G and H).

To further explore the regulation of *Sema3c*, we assessed the role of Tbx1, a downstream target of Foxc1 and Foxc2^16–18^. Tbx1 is expressed in the SHF, but not in cNCCs^19–21^, and is a major genetic modulator of cardiac OFT defects in 22q11.2 deletion syndrome (22q11DS)^22–24^. A previous study showed that *Tbx1* null mice showed a significant reduction in *Sema3c* expression in the OFT region^25^, though the exact regulatory mechanism of Tbx1 on *Sema3c* is unclear. We investigated the role of Tbx1 on the OFT enhancer in the *Sema3c* 5′-flanking region and found that Tbx1 itself has no effects on the transcriptional activity of the OFT enhancer region (Supplementary Figure S1). However, overexpression of Tbx1 together with Foxc1 showed a significant increase in the transcriptional activation of the OFT enhancer, whereas overexpression of Tbx1 with Foxc2 showed milder synergistic interactions than with Foxc1. (Supplementary Figure S2A). Furthermore, Foxc1 displayed a direct interaction with Tbx1 in the co-immunoprecipitation assay (Supplementary Figure S2B). As there is no Tbx1-specific binding locus on the enhancer region, these findings suggest that Tbx1 acts as a transcriptional co-factor of Foxc1 and Foxc2 and promotes synergistic *Sema3c* expression in the OFT myocardium derived from SHF progenitor cells. Taken together, these results indicate that Foxc1 and Foxc2 can directly regulate *Sema3c* expression in the OFT through the Fox-binding site on the 5′-flanking sequence of *Sema3c*, and that Tbx1 synergistically enhances the function of Foxc1 via direct interactions and partially enhances Foxc2 indirectly during cardiac development.

### Ectopic expression of Sema3c is associated with cardiac neural crest migration defects in Tbx1 hypomorphic mice

**Figure 2:**
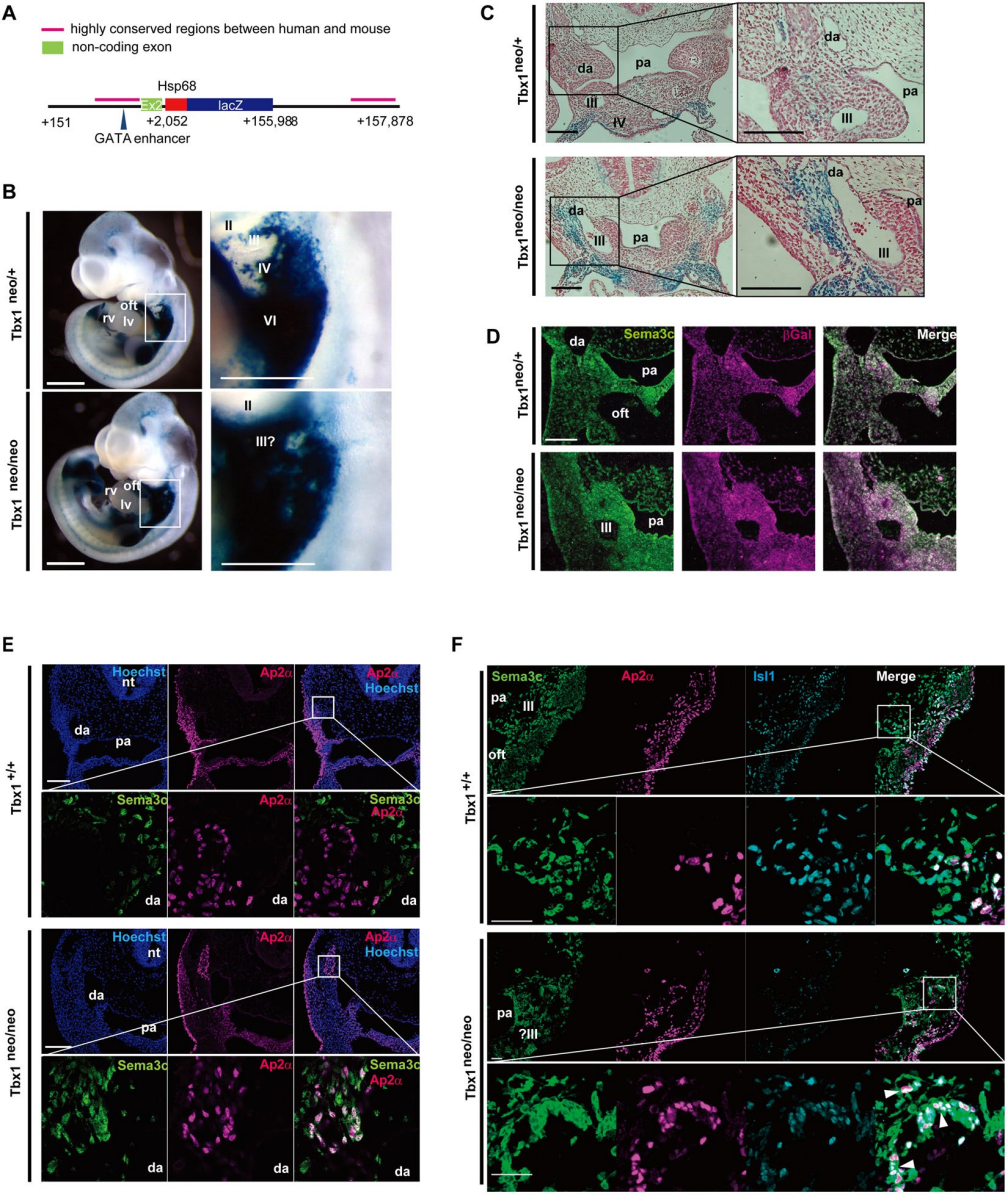
Altered expression of Sema3c and defects of cardiac neural crest migration in the hypomorphic state of *Tbx1* (*Tbx1* ^neo/neo^). (**A**) *Sema3c* int1/3′-*lacZ* transgene construct with *Sema3c* intron1, exon2, and the 3′-flanking sequence including highly conserved regions (indicated by black bar) (from +151 to +2052 followed by the *hsp68* basal promoter, *lacZ* gene, and the 3′ region from +155988 to +157878). (**B**) LacZ expression in *Sema3c* int1/3′-*lacZ*: *Tbx1* ^neo/+^ (upper, left) and *Tbx1* ^neo/neo^ (lower, left) double transgenic (tg) embryos at E10.5 reminiscent of endogenous Sema3c mRNA expression. High magnification views of the pharyngeal arch region (white box) are shown in the right lane. Enhanced lacZ expression in the pharyngeal arch region of *Sema3c* int1/3′-*lacZ* tg:*Tbx1* ^neo/neo^ embryo (lower, right) compared to *Sema3c* int1/3′-*lacZ* tg:*Tbx1* ^neo/+^ embryo (upper, right) Scale bars, 500 μm. (**C**) Coronal section in the pharyngeal arch region of *Sema3c* int1/3′-*lacZ* tg:*Tbx1* ^neo/neo^ or *Sema3c* int1/3′-*lacZ* tg:*Tbx1* ^neo/+^ embryos. Right panels are high magnification views of boxed areas in the left panels. Scale bars, 100 μm. (**D**) Immunostaining for Sema3c (green) and βGal (red) on coronal sections of the pharyngeal arch region in *Sema3c* int1/3′-*lacZ* tg:*Tbx1* ^neo/+^ or *Sema3c* int1/3′-*lacZ* tg:*Tbx1* ^neo/neo^ embryos. Scale bars, 100 μm. (**E**) Immunostaining for nucleus (blue), Sema3c (green) and Ap2α (red) on coronal sections of the pharyngeal arch region in wild-type (*Tbx1* ^+/+^) and *Tbx1* ^neo/neo^ mutant embryos at E10.5. Lower panels show hyper-magnified views of areas indicated by the white box in the upper panels. Scale bars, 100 μm. **(F)** Immunostaining for nucleus (blue), Sema3c (green), Ap2α (red) and Isl1 (cyan) on coronal sections of the pharyngeal arch region in wild-type (*Tbx1* ^+/+^) and *Tbx1* ^neo/neo^ mutant embryos at E10.5. Cut-in panels showed hyper-magnified views of cells indicated by white arrowheads in the upper panels. Scale bars, 50 μm: lower magnification view; 10 μm: hyper magnification view. lv, left ventricle; rv, right ventricle; oft, outflow tract; pa, pharynx; nt, neural tube; da, dorsal aorta; II, III, IV, and VI, 2nd, 3rd, 4th, and 6th pharyngeal arch arteries, respectively.

We further validated the role of Tbx1 in the regulation of *Sema3c* expression using *Tbx1* hypomorphic mice^26^. In a manner similar to a previous study with Tbx1 knockout mice^25^, Sema3c expression was reduced in the OFT region of *Tbx1* hypomorphic mice (Supplementary Figure S3: *Tbx1* ^neo/neo^)^26^ compared to that in heterozygous littermates (Supplementary Figure S3: *Tbx1* ^neo/+^). Interestingly, mice hypomorphic for *Tbx1* showed ectopic Sema3c expression in the pharyngeal arches region at E10.5 in comparison with wild-type mice (Supplementary Figure S3). Since lacZ transgene regulated by Sema3c intron 1 or 3′-flanking region showed βGal expression in pharyngeal arch region (Supplementary Figure S4)^13^, we generated a transgenic mouse line harboring the intron1 and the 3′-flanking genomic sequence of *Sema3c* (*Sema3c* int1/3′-*lacZ* tg; Fig. 2A) and crossed it into the *Tbx1* hypomorphic background. LacZ expression in *Sema3c* int1/3′-*lacZ* transgenic (tg) embryos was consistent with endogenous Sema3c mRNA expression at E10.5, whereas lacZ expression was detected in large part of pharyngeal arch region in Tbx1 hypomorphic embryos (Fig. 2B). Coronal sections revealed that the lacZ was ectopically expressed in pharyngeal arches in *Tbx1* hypomorphic embryos (Fig. 2C). βGal expression was coincident with the Sema3c expression pattern in the pharyngeal arch region as observed by immunofluorescence, both in wild-type and *Tbx1* hypomorphic embryos (Fig. 2D). In wildtype embryos at E10.5, Sema3c was expressed in the mesoderm of the pharyngeal arch region, but not in cNCCs labeled with anti-AP2α antibody (Fig. 2E: Tbx1^+/+^). Intriguingly, in *Tbx1* hypomorphic embryos at E10.5, cNCCs migrated less toward the ventral area of the pharyngeal arch region and aggregated around the dorsal arteries with remarkable ectopic expression of Sema3c, as demonstrated by the overlapping expression of Sema3c and AP2α (Fig. 2E: *Tbx1* ^neo/neo^). Moreover, at this stage, Sema3c was highly expressed in the Isl1-positive SHF progenitor cells in the wild-type embryos; in contrast, Sema3c was expressed in AP2α-positive cNCCs in the pharyngeal arch region along with a significant decrease of Isl1-positive cells due to developmental defects of SHF progenitor cells in *Tbx1* hypomorphic embryos (Fig. 2F). A past study has shown that Sema3c attracts cNCCs and regulates their migration into the most proximal part of the OFT^27^. To test whether the abnormal upregulation of Sema3c may disturb cNCC migration, we utilized the primary culture of cNCCs and found that lentiviral-mediated Sema3c overexpression caused aggregation of cNCCs that could be rescued by Sema3c neutralizing antibodies (Supplementary Figure S5). These results suggest that Tbx1 expression in the SHF is essential to inhibit the ectopic expression of Sema3c in cNCCs migrating in the pharyngeal arch region, and that loss of Tbx1 function may lead to ectopic expression of Sema3c in cNCCs, which results in their abnormal migration and/or aggregation.

### Sema3c is negatively regulated by Fgf8, a downstream target of Tbx1, during the migration of cardiac neural crest cells

**Figure 3:**
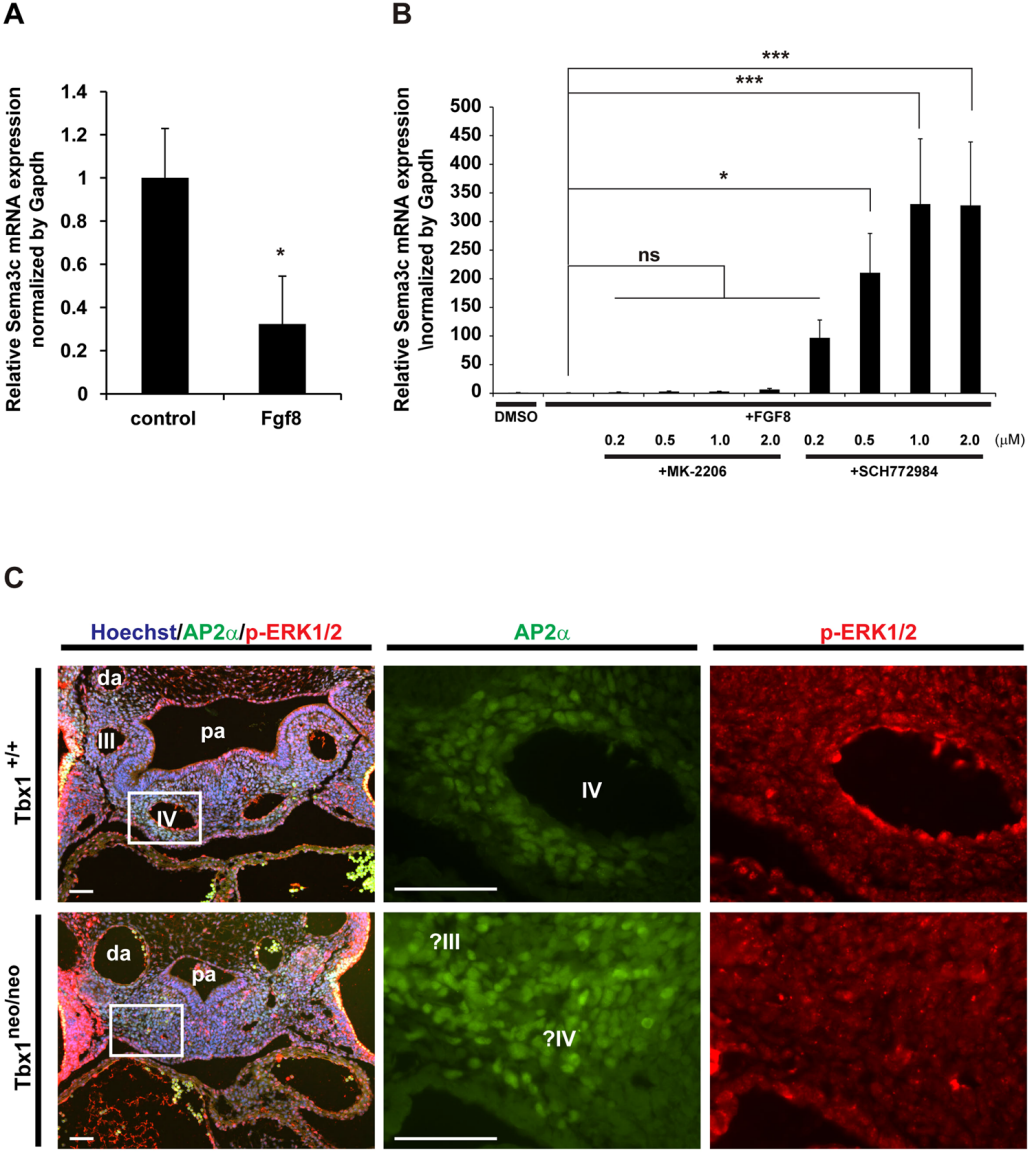
Sema3c expression is negatively regulated by Tbx1-Fgf8-Erk signaling. (**A**) Sema3c mRNA expression in cNCC explants treated with or without Fgf8 for 3 days. n = 5 per group. (**B**) Sema3c mRNA expression in cNCC explants treated with Fgf8 with or without AKT inhibitor (MK-2206) or ERK1/2 inhibitor (SCH772984). n = 6 per group. (**C**) Immunostaining for phosphor-Erk1/2 (red) and Ap2α (green) of the pharyngeal arch region in wild-type (*Tbx1* ^+/+^) and *Tbx1* ^neo/neo^ mutant embryos at E10.5. Right panels showed hyper-magnified views of area indicated by the white box on left panels. Scale bars, 100 μm. pa, pharynx; da, dorsal aorta; III and IV, 3rd and 4th pharyngeal arch arteries. *p < 0.05, ***p < 0.005. Error bars represent s.e.m.

Given the importance of Tbx1 in OFT development, it may also regulate the expression of *Sema3c* in cNCCs. However, the expression of Tbx1 in the SHF and its absence in cNCCs^16, 20^ suggests the existence of some secreted factors from the SHF, downstream of Tbx1, which mediate the inhibitory regulation of Sema3c in cNCCs. Using a candidate approach, we focused on Fgf8 signaling that is considered a downstream target of Tbx1 or Foxc genes^17, 26^. Fgf8 is a secreted signaling protein expressed and functioning in the cardiac crescent and splanchnic mesoderm as well as in the pharyngeal endoderm and ectoderm during development of the pharyngeal arches and the OFT^28–31^. In the pharyngeal arch region, Fgf8 is thought to be secreted from SHF progenitor cells and may contribute to the interaction between SHF and cNCCs^26^. Furthermore, a recent report showed that Fgf8 was both chemotactic and chemokinetic for cNCCs^32^. To elucidate the role of Fgf8 in regulation of Sema3c expression during the development of cNCCs, we utilized the primary culture of cNCCs. Interestingly, Sema3c expression in primary cultured cNCCs was downregulated by treatment with Fgf8 (Fig. 3A). It has been reported that Fgf8 signaling transduced the signal via ERK and AKT pathway and that the ERK signaling contributed to cNCC migration^32–34^. The inhibition of ERK signaling caused significant upregulation of Sema3c in Fgf8-stimmulated cNCCs whereas inhibition of ALK signaling showed no effect on Sema3c expression (Fig. 3B). Furthermore, the phosphorylation of ERK1/2 was disturbed in cNCCs around the pharyngeal arch arteries of Tbx1 hypomorphic embryos as compared to wild-type littermates (Fig. 3C). These results indicate that Tbx1-Fgf8-ERK signaling negatively regulates the expression of Sema3c in cNCCs.

To validate the role of Fgf8 in the differentiation of cNCCs during cardiogenesis, we utilized a Wnt1-Cre/floxed-EGFP transgenic mouse line^35, 36^. Slight mechanical dissociation of the third through sixth pharyngeal arch regions in the Wnt1-Cre/floxed-EGFP transgenic mice resulted in the formation of EGFP-positive neural spheres (Supplementary Figure S6A). After seven days of incubation in differentiation media, the EGFP-positive neural spheres gave rise to multi-lineage derivatives including neurons, glia, and smooth muscle cells (Supplementary Figure S6B). This suggests that neural spheres derived from roughly the third to sixth pharyngeal arch regions maintain multi-lineage potential. However, the addition of Fgf8 did not affect the differentiation efficiency of cNCCs to the three lineage derivatives (Supplementary Figure S6C), suggesting that Fgf8 does not play a role in the differentiation of cNCCs.

**Figure 4:**
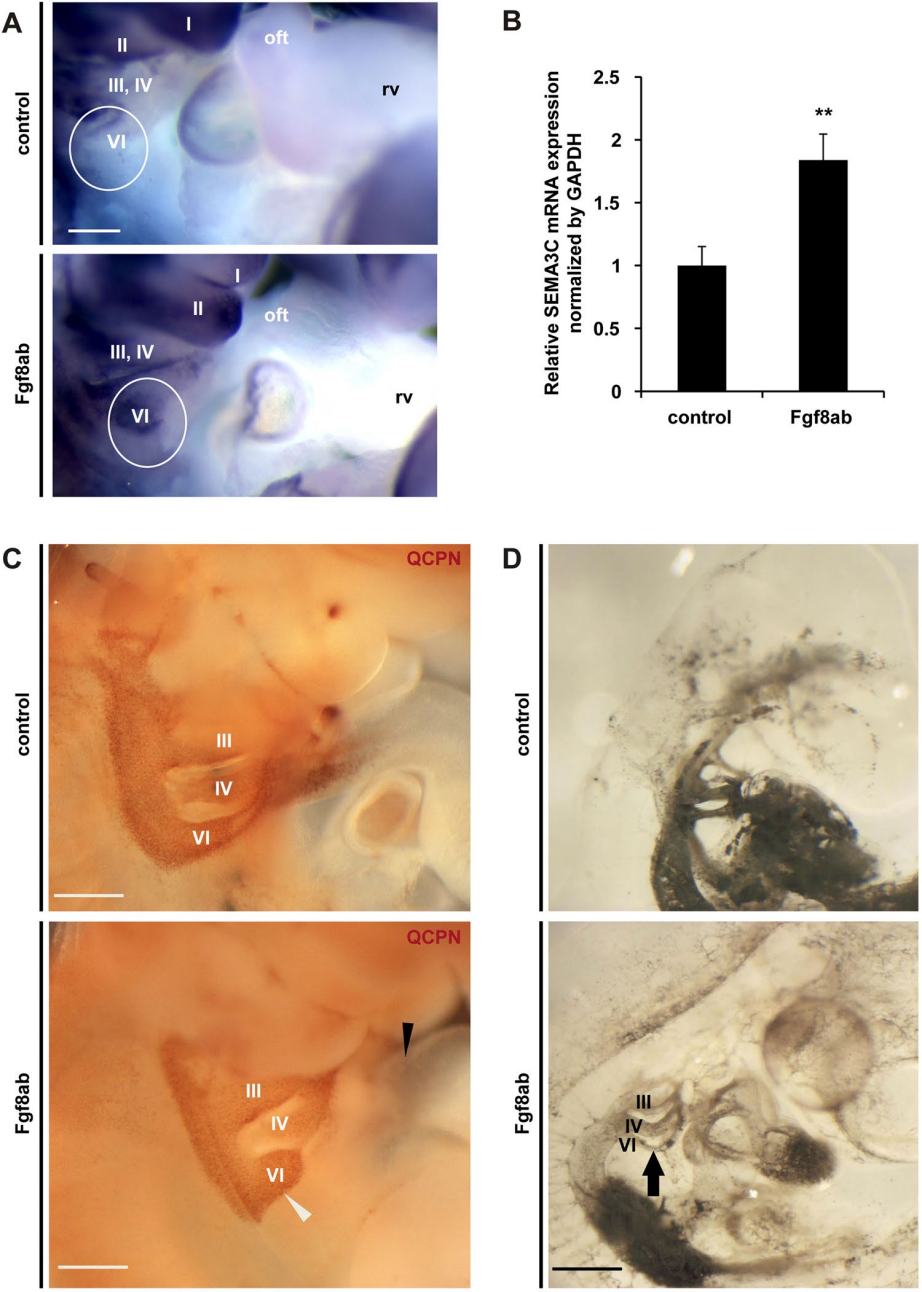
*In ovo* blocking of the Fgf8 signal causes abnormal Sema3c expression and migration defects in cardiac neural crest cells. (**A**) Injection of Fgf8 neutralizing antibody (Fgf8ab) led to abnormal ectopic enhancement of *SEMA3C* expression in the pharyngeal arch region (white circle) in HH22 chick embryos. (**B**) SEMA3C mRNA expression in the pharyngeal arch region of HH22 chick embryos (control) and those injected with Fgf8ab (n = 6 per group). (**C**) Pharyngeal arch regions of chick-quail chimeras with bilateral transplantation of cNCCs. Fgf8ab injection causes less migration of cNCCs from the 6th pharyngeal arch region (white arrowhead) to the outflow tract (black arrowhead) compared to control. Thirteen chimeras and thirteen control embryos were analyzed. (**D**) India ink injection demonstrated hypoplasia of the 6th pharyngeal arch artery in Fgf8ab injected embryos (arrow) compared to control. v, ventricle; oft, outflow tract; III, IV and VI, 3rd, 4th, and 6th pharyngeal arch arteries, respectively; Paa, pharyngeal arch arteries. Scale bars, 500 μm. **p < 0.01. Error bars represent s.e.m.

To test whether blockage of Fgf8 signaling could cause migration defects in cNCCs with enhanced expression of SEMA3C, we injected Fgf8 neutralizing antibody between the 4th and the 6th pharyngeal arches in chick embryos at HH stage 17 *in ovo*. After 48 hrs incubation, embryos injected with Fgf8 neutralizing antibody showed the ectopic expression of SEMA3C mRNA surrounding the 4th to 6th pharyngeal arches (Fig. 4A and B). Furthermore, chick-quail chimera analyses demonstrated that embryos injected with Fgf8 neutralizing antibody resulted in migration defects of cNCCs at HH stage 22 (Fig. 4C). Consistently, ink injections revealed hypoplasia of the 6th pharyngeal arch artery in chick embryos injected with Fgf8 neutralizing antibody compared to control embryos (Fig. 4D). Taken together, these results suggest that Tbx1-Fgf8 signaling inhibits the expression of SEMA3C in migrating cNCCs, resulting in proper guidance of their migration from the dorsal pharyngeal region to the OFT.

## Discussion

In this study, we demonstrate a molecular mechanism for development of proper OFT septation, where Sema3c is tightly regulated in a spatio-temporal fashion during the interaction between cNCCs and SHF. *Sema3c* expression in the OFT myocardium was shown to be directly regulated by Foxc1 and Foxc2 through conserved Fox-binding sites, where Tbx1 acts synergistically as a co-factor for Foxc proteins. Partial downregulation of *Sema3c* was consistently observed in *Foxc1*- or Foxc2 null embryos; this is probably because of their redundancy, as a previous report showed that Foxc1 and Foxc2 played similar dose-dependent roles in development of the OFT and aortic arch system^37^. We and others have previously shown that Foxc proteins also regulate the expression of Tbx1 in the SHF in a dose-dependent manner^16–18^. It was reported that the OFT defect resulting from disruption of the Sema3c locus in mice was reminiscent of that seen in mice with ablation of Tbx1^25^, whereas the compound Foxc1/Foxc2 mutant mice showed severe hypoplasia in outflow tract and right ventricle^17^. These findings may suggest the essential upstream effect of Foxc1/Foxc2 on multiple factors in the OFT development, including Tbx1-Sema3c transcriptional cascade.

Proper cNCC migration is necessary for the septation of great arteries. Although genetic ablation studies have shown many potential factors involved in the development of cNCCs^38^, the mechanism for proper homing of cNCCs from the neural tube to the OFT is still largely unknown. Plein *et al*. reported that mice with Sema3c ablations in the neural crest showed disrupted septation in the OFT, although the mesenchymal cells were located in the OFT cushion^39^. These mesenchymal cells in the OFT cushion lacked *Sema3c* expression; however, expression was maintained in the proximal region of the OFT myocardium. It was also reported that Sema3c attracted cNCCs and regulated their migration to the extreme proximal region of the OFT^27^. In this study, we showed that downregulation of Tbx1 resulted in dysregulated expression of *Sema3c* in the pharyngeal arch region, causing abnormal aggregation and migration of cNCCs. We confirmed that the overexpression of *Sema3c* in cNCCs led to their aggregation *in vitro*. On the other hand, *Sema3c* was downregulated in the OFT region of Tbx1 hypomorphic mice; this observation is consistent with that of a previous report^25^. Taken together, these findings suggest a developmental model that Sema3c from the SHF-derived OFT myocardium plays a role as an attractant for cNCCs, that inhibition of *Sema3c* is necessary in cNCCs during their migration, and that the activation of *Sema3c* is required for normal aggregation of cNCCs in the proximal OFT region. Proper spatio-temporal expression of *Sema3c* may be essential for correct septation of the OFT.

Given the close apposition and function of cNCCs and the SHF in the development of the OFT, it is possible that behavior of cNCCs may be dependent on signals from the SHF. As for interaction of the two cardiac stem cell lineages, our findings suggest that Fgf8 from the SHF may diffuse distally to directly influence the development of cNCCs via activation of ERK signaling. A recent report showed that Fgf8 was both chemotactic and chemokinetic for cNCCs^32^. Fgf8 was also shown as a downstream effector of Tbx1 secreted from the SHF in the pharyngeal arch and OFT region^26^. The ablation of *Fgf8* with a *Tbx1Cre* transgene resulted in OFT defects^40^. Suppression of ERK signal in zebrafish causes non-migration of cNCCs^41^. Taken together, our data suggest that inhibition of Sema3c in cNCCs by Fgf8 secreted from the SHF in regulation by Tbx1 may contribute to a proper condensation of the cNCC cluster in the OFT that establishes the septum.

**Figure 5:**
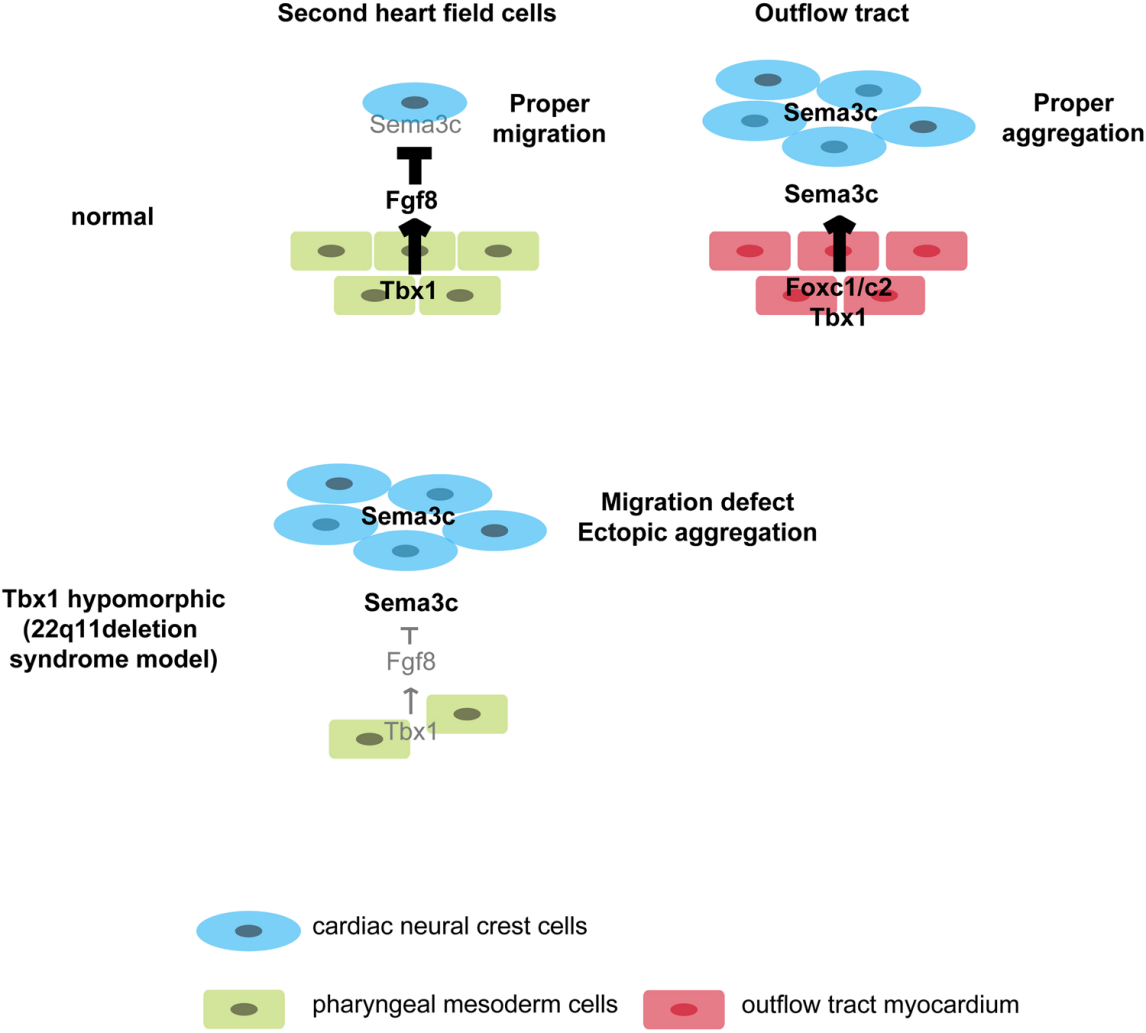
A working model for the regulation of *Sema3c* in developing cardiac neural crest cells (cNCCs) and second heart field (SHF) progenitor cells. During normal development, Tbx1 may restrict the expression of *Sema3c* in the SHF along the pharyngeal arch region by blocking ectopic *Sema3c* expression in cNCCs via Fgf8 signaling. Foxc1, Foxc2, and Tbx1 may then synergistically activate the expression of *Sema3c* in the SHF during migration into the outflow tract myocardium and differentiation. In contrast, in *Tbx1* hypomorphic mice, downregulation of Tbx1-Fgf8 signaling in the pharyngeal arch region may lead to misexpression of *Sema3c* in migrating cNCCs, resulting in their aggregation and the disruption of migration.

In conclusion, this study implicates Foxc1, Foxc2, and Tbx1-Fgf8 signaling as essential regulators for Sema3c expression, contributing the temporal and spatial regulation in the development of cNCCs during OFT and aortic arch formation. We propose a model molecular mechanism in which Tbx1 restricts the expression of Sema3c in the SHF by blocking its ectopic expression in migrating cNCCs via Fgf8 signaling in the pharyngeal arch region during development of the OFT (Fig. 5). Foxc1 and Foxc2, together with Tbx1, may synergistically activate Sema3c expression in progenitor cells in the SHF in a process during their migration and differentiation into the OFT myocardium. This orchestrated Sema3c regulation may lead to a signal for the correct migration of cNCCs into the OFT to give rise to the septum. Our findings provide new insight into the interaction between cNCCs and the SHF, as well as into the etiology of congenital heart diseases involving OFT defects.

## Methods

### Mice

*Tbx1* ^*neo/+*^ mice were kindly provided by Dr. Deepak Srivastava^26^. Foxc1^-/-^ and Foxc2^-/-^ mice embryos were kindly provided by Dr. Tsutomu Kume^37^. Transgenic mice expressing Cre recombinase under control of the Wnt1 promoter/enhancer (Wnt1-Cre)^35^ were mated with EGFP reporter mice (CAG-CAT-EGFP)^36^ to obtain Wnt1-Cre/Floxed-EGFP double-transgenic mice. Adult wild-type mice were purchased for mating from Sankyo Labo Service (Tokyo, Japan). PCR analysis of tail and yolk sac DNA of the *Tbx1-neo allele*, *foxc1 null and foxc2 null alleles* have been previously described^26, 37^. All experimental procedures were approved by the ethics committee of Keio University and were in accordance with the Guide for the Care and Use of Laboratory Animals (U.S. National Institutes of Health).

### Plasmid construction and site-directed mutagenesis

Mouse *Sema3c* 5′-flanking regions (for mouse Sema3c promoter/enhancer-*lacZ* plasmids; pm-691, pm-492 and pm-492m) or intron1 and 3′-flanking regions (for mouse *Sema3c* int1/3′-*lacZ* tg plasmid) were generated from the DNA extract of mouse tail by PCR and different fragments of the *Sema3c* upstream, intron1 or 3′-flanking regions were once cloned into plasmid pBluescript (Stratagene), then subcloned into the *hsp68lacZ* reporter construct^42^ or pGL3-Basic (Promega, Madison, WI, USA). The PCR-based mutagenesis on this plasmid was performed using *pfu* DNA polymerase and a QuikChange site-directed mutagenesis kit (Stratagene). The primers used were 5′-GATTCAGTGTGGAAAGGTggGCATAATTACTGATTTCAG-3′ and 5′-CTGAAATCAGTAATTATGCccACCTTTCCACACTGAATC-3′. Restriction mapping and sequence analysis confirmed the presence of the mutation, and the insert was excised and cloned into the *hsp68lacZ* reporter plasmid. All constructed vectors were verified by sequencing.

### Production and analysis of transgenic mouse embryos

DNA fragments were isolated from mouse *Sema3c* promoter/enhancer–*lacZ* plasmids (pm-691, pm-492 and pm-492m) or mouse *Sema3c* int1/3′-*lacZ* tg plasmid using *Sal*I site. These fragments were microinjected into fertilized oocyte pronuclei, eggs were transferred into the oviducts of pseudopregnant females, and transgenic embryos were identified by PCR analysis for each promoter or enhancer–*lacZ* gene in yolk sac DNA. Embryos were harvested at E10.5 and stained for beta-galactosidase activity, as described previously^13^.

### Embryo culture and neutralizing antibody injection

Fertilized chicken eggs were incubated at 38 °C in a humidified incubator until the embryos reached stages HH17^43^. The eggs were windowed and prepared for following procedures. Goat anti-Fgf8 polyclonal antibody (Santa Cruz sc-6958) were diluted by phosphate-buffered saline (PBS(−)) to the concentration of 100 μg/mL. The neutralizing antibody or PBS(−) (for control) was injected into pharyngeal arch region between 4th and 6th pharyngeal arch arteries of HH17 chick embryos. The treated embryos were cultured for 48 hrs at 38 °C, then dissected and fixed with 4% PFA.

### Ink Injections

India ink (Rotring, Germany or Kiwaguro, Sailor, Japan) was injected into a vitelline vein using a capillary pipette pulled to a fine diameter. The ink was ejected using gentle, positive pressure until it filled the intravascular compartment. The embryos were dissected from surrounding membranes and immersion fixed in neutral buffered formalin overnight, dehydrated, and cleared in benzyl benzoate:methylsalicylate (1:1).

### Quail-Chick chimera Preparation

Fertilized chicken eggs and *Cortunix japonica* quail eggs were used. They were incubated at 38 °C in forced-draft incubators with a constant humidity until the embryos reached stages HH8–10. The eggs were windowed and prepared for microsurgery following the procedures previously described^44, 45^. The transplantation procedure always used chick embryos as hosts and quail embryos as donors, and was performed homotopically. Each presumptive arch region (3rd, 4th and 6th) of the cardiac neural crest was transplanted individually. Bilateral transplantations were done. India ink, diluted 1/10 in saline solution, was injected into the subgerminal cavity to allow visualization of the embryo. The vitelline membrane was torn over the area of cardiac neural crest to be excised. The neural folds containing premigratory neural crest cells from a chick embryo was removed at a defined level by using an electrolytically sharpened tungsten needle. The region was replaced with the corresponding neural folds from a quail embryo. After the microsurgery, the egg-shell windows were sealed by using cellophane tape and the eggs were returned to the incubator. Sham-operated embryos were produced by tearing the vitelline membrane with no further surgery. Control embryos were opened the window and counted the somite with no treatment. 100 μg/mL of goat anti-Fgf8 polyclonal antibody (Santa Cruz) or PBS(−) (for control) was injected into pharyngeal arch region between 4th and 6th pharyngeal arch arteries of HH17 chimera embryos. The treated embryos were cultured for 48 hrs at 38 °C, then dissected and fixed with 4% PFA.

### Chick RNA extraction

HH17 chicken embryos were harvested and the pharyngeal arch region between 3rd and 6th pharyngeal arch arteries was dissected out with resection of the heart. Pharyngeal arch region were collected into 1.5 ml tubes with 1 ml of Trizol reagent (Invitrogen) and immediately frozen in liquid nitrogen and stored at −80 °C. Total RNA extraction was performed according to the manufacturer’s instructions.

### Immunohistochemistry for Quail-Chick chimera embryos

For whole-mount immunostaining, embryos were fixed in 4% paraformaldehyde in PBS at 4 °C for 1 hr and rinsed in PBS at room temperature. The embryos were dehydrated in a graded series of methanol and stored at −20 °C. Endogenous peroxidase activity was blocked with 6% H_2_O_2_ in methanol for 6 hrs at room temperature and the embryos were rehydrated and blocked in PBSMT (2% non-fat milk, 1.0% Triton X-100, PBS) for 1 hr twice at room temperature. The embryos were incubated with monoclonal antibody QCPN (Developmental Studies Hybridoma Bank), which targets a nuclear antigenic determinant that is specific to quail cells, in PBSMT for 2 days at 4 °C while being agitated on a shaking platform. After extensive washes with PBSMT at 4 °C six times (1 hour each), the embryos were incubated with horseradish peroxidase (HRP)-conjugated goat anti-mouse IgG (Jackson ImmunoResearch Lab Inc, USA) in PBSMT (1:200 dilution) at 4 °C overnight. Embryos were rinsed in PBSMT at 4 °C six times (1 hour each) and finally in PBST (1% Triton X-100, PBS) for 20 minutes at room temperature. The peroxidase staining was performed by incubating embryos in 0.16 mg/ml 3-3′-diaminobenzidine tera-hydrochroride (DAB, Sigma) in PBT for 1 hr followed by the addition of H_2_O_2_ to the final concentration of 0.01%. The staining reaction was stopped by rinsing in PBT. The embryos were postfixed in 4% paraformaldehyde in PBS at 4 °C for 1 hr. For sectioning, embryos were fixed in 4% paraformaldehyde in PBS at 4 °C overnight and rinsed in PBS and dehydrated in graded alcohols, cleared, and embedded in paraffin following the procedures previously described^45^. Sections were cut at 10 μm and deparaffinized, rehydrated, blocked with 0.3% H_2_O_2_ in PBS. The sections were blocked with 2% bovine serum albumin in PBS and incubated with QCPN overnight at 4 °C. After washing, they were incubated with biotin-conjugated goat anti-mouse IgG (Vector BA-9200) diluted 1:200 in 2% bovine serum albumin in PBS. The slides were incubated with ABC Elite kit (Vector) and then washed. After final washing, the slides were visualization with DAB.

### RT-PCR and real-time quantitative PCR

Total RNA was extracted using the Trizol reagent (Invitrogen), and RT-PCR was performed as described previously^46^. For quantitative analysis of mouse *Sema3c* and chicken SEMA3C expression, the respective cDNA was used as the template in a TaqMan real-time PCR assay using the ABI Prism 7500 FAST sequence detection system (Applied Biosystems) according to the manufacturer’s instructions. All samples were run in triplicate. The data was normalized to mouse *Gapdh* or chicken SEMA3C expression. The TaqMan probes for mouse *Sema3c*, *Gapdh*, and chicken *SEMA3C* and *GAPDH* were Mm00443121_m1, Mm99999915_g1, Gg03364166_m1 and Gg03346984_g1, respectively.

### *In Situ* Hybridization

Whole-mount *in situ* hybridization analyses of mouse and chick embryos were performed as described^25^. The mouse Sema3c and chicken SEMA3C RNA probes were used for *in situ* hybridization. RNA transcripts were synthesized using a digoxigenin (DIG)-labeling kit (RocheMannheim) according to the manufacturer’s protocol.

### Immunohistochemistry for mouse embryos

Mice embryos were fixed in 4% paraformaldehyde on ice for 1 hr and embedded in OCT, frozen in dry iced hexane, and kept at −80 °C until sectioned. Frozen sections (8 μm) were blocked with 5% goat serum in PBS with 0.1% Tween20 for 1 hr at room temperature and stained with following primary antibodies: rat monoclonal anti-Sema3 (1:50, R&D MAB1728), mouse monoclonal anti-AP2α clone 3B5 (1:100, Developmental studies hybridoma bank), rabbit polyclonal anti-β-galactosidase (1:1000, Rockland 200–4136) and rabbit polyclonal anti-Isl1 (1:100, Abcam ab20670). For phosphor-Erk1/2 staining, mice embryos were fixed in 4% paraformaldehyde on ice for 1 hr and embedded in paraffin. Paraffin sections (7 μm) were quenched by 3% H_2_O_2_ in methanol and were treated for 20 min with Citrate Buffer, pH 6.0 (Sigma-Aldrich) using standard protocols. Samples were blocked with 5% goat serum in PBS with 0.1% Tween20 for 1 hr at room temperature and stained with rabbit monoclonal anti-phospho-ERK1/2 clone D13.14.4E (1:100, Cell Signaling #4370) and mouse monoclonal anti-AP2α Clone 3B5 (1:40). Signals were amplified using TSA Biotin Systems (Perkin Elmer NEL700A001KT). Secondary antibodies were used as follows: anti-rabbit IgG (Alexa 647 A-21245), anti-mouse IgG (Alexa 555 A-21422), anti-mouse IgG (Alexa 488 A-11001), anti-rabbit IgG (Alexa 555 A21429), and anti-rat IgG (Alexa 488 A-11006, all from Molecular Probes), Biotinylated Goat anti-rabbit IgG (Vector BA-1000) and Streptavidin, Alexa Fluor 594 conjugate (Molecular Probes S-11227). Nuclear counterstaining was performed with Hoechst 33342 (10 mg/ml, Sigma B2261). Image acquisition was performed on a universal fluorescence microscope (Axioskop 2 Plus; Carl Zeiss) and a confocal microscope (LSM 510 Meta; Carl Zeiss) and ZEN software (Carl Zeiss).

### Primary culture of mouse cNCCs

Pregnant wild type C57BL6 female mice were deeply anesthetized and sacrificed by cervical dislocation and embryos were collected at E9.5 into dissection medium. The neural tubes between the otic vesicles and sixth somites were cut out. The neural tubes were incubated in 1 mg/mL of collagenase/dispase (Sigma-Aldrich) in PBS buffer at room temperature for 10 minutes followed by removal of non-neural ectoderm and mesoderm tissues. The isolated neural tubes were placed into the wells of fibronectin (Sigma-Aldrich)-coated 24-well plates and cultured with Self-Renewal (SR) medium consisting of 12.5 mL low glucose DMEM (GIBCO), 7.5 mL Neurobasal Medium (GIBCO), 117 μM retinoic acid (Sigma-Aldrich), 50 mM 2-mercaptoethanol (GIBCO), 3.75 mL Chick Embryo Extract (MP Biomedicals), 250 μL N2 salt supplement (GIBCO), 500 μL B27 supplement (GIBCO), 250 μL penicillin-streptomycin (GIBCO), 20 μg/mL human recombinant IGF1 (R&D #291-G1) and 20 μg/mL human recombinant bFGF (Pepro Tech #100–18b)^47^. After 24 hrs incubation at 37 °C, the neural tubes were removed.

### Lentiviral mediated Sema3c overexpression assay

For lentiviral vector production, whereby 80% confluent 293T cells (Thermo Fisher Scientific) were transfected overnight with virus production vectors including pLenti-c-Myc-DKK Sema3c (Origene) or pLenti6-EGFP (control vector), pucMDG, and pCMVΔ8.91^48^ using Lipofectamine 2000 (Life technologies) and were subsequently purified, concentrated using Lenti-X Concentrator (Clontech), and frozen until use. Titers were measured by using qPCR Lentivirus Titration Kit (Applied Biological Materials) and *Power* SYBR Green PCR Master Mix (Applied Biosystems). For transduction, concentrated viral supernatant containing 7.5 × 10^6^ transducing particles were added into the culture medium of cNCC for 24 hrs with or without 1 μg/mL rat anti-Sema3c monoclonal antibody (R&D MAB1728), upon which cNCCs’ phenotype was observed using a Leica DM IL LED microscope (Leica).

### Small molecule treatments

Mouse cNCCs isolated from neural tubes were seeded to fibronectin-coated 6 well plates and cultured in SR medium with 100 ng/mL human/mouse recombinant FGF8 (R&D) with or without ERK1/2 inhibitor (SCH772984 Selleck Chemicals) or AKT inhibitor (MK-2206 Selleck Chemicals) for 3 days.

### Sphere-Forming Culture

Pregnant Wnt1-Cre/Floxed-EGFP mice or wild-type C57BL6 mice were deeply anesthetized and sacrificed by cervical dislocation, and embryos were collected at E9.5 into PBS with 1% glucose. The pharyngeal arch region between 3^rd^ and 6^th^ pharyngeal arch arteries was dissected and removed with resection of the neural tube and heart. The samples were then collected into 1.5 ml tubes with 1 ml of a serum-free sphere-forming medium consisting of MHM medium [DMEM/F-12 (1:1) (GIBCO 12100-046/21700-075) supplemented with insulin (25 mg/ml), transferrin (100 mg/ml), progesterone (20 nM), sodium selenate (30 nM), putrescine (60 nM) (all from Sigma-Aldrich)], recombinant human EGF (100 ng/ml) (Pepro Tech #100-15), human FGF-basic (100 ng/ml) (Pepro Tech #100-18b), and B27 (20 ng/ml) (Invitrogen). The dissected tissue was mechanically dissociated by pipetting, and cells from the pharyngeal arches were seeded at 5 × 10^3^ cells/ml into an ultra - low attachment 6-well plate (Corning) with serum-free sphere-forming medium^46^ and incubated at 37 °C, with 5% CO_2_; half of the medium was changed after 4 days.

### Differentiation Analysis

Spheres were plated on poly-D-lysin/laminin (Sigma P7405/Invitrogen 23017-015)-coated 8-well chamber slides (Iwaki 5732–008) for immunostaining and cultured for 7 days in the following differentiation medium: MHM medium with 1% FBS (Sigma-Aldrich) and 25 ng/mL mouse Fgf8 (Peprotech) or PBS (control).

### Immunocytochemistry

Cells were fixed in 4% PFA and stained with the following primary antibodies: anti- β-III tubulin (mouse IgG2b, 1:500, Sigma T8660), anti- αSMA (mouse IgG2a, 1:1000, Sigma A2547), and anti-GFAP (rat IgG, 1:500, Invitrogen 13-0300). The secondary antibodies used for staining were the following: anti-mouse IgG2b (Alexa 488 A-21141), anti-mouse IgG2a (Alexa 350 A-21130), and anti-rat IgG (Alexa 555 A21434). The stained samples were observed under a universal fluorescence microscope (Axioskop 2 Plus; Carl Zeiss).

### ChIP assay

For the *in vivo* ChIP experiments, extracts were prepared from approximate 1 × 10^7^ of P19CL6 cells^49^ which were transfected by FLAG-tagged Foxc1 or Foxc2 expression vectors 48 hours before harvest. For the ChIP assays, the ChIP-IT Express Chromatin Immunoprecipitation Kits (Active Motif) were used according to manufacturer’s protocol. Primers in PCR reactions were 5′-ACAAGCTTCGCTGGACTGAT-3′ and 5′-TCTCGGCTCACTCTCTGGTT-3′ (−148 + 43). The amplified region corresponded to the mouse *Sema3c* promoter, which encompasses the FOX-binding site.

### Co-immunoprecipitation assays

COS1 cells were transiently transfected by FLAG-tagged Foxc1/Foxc2 or Myc-tagged Tbx1 expression vectors with Lipofetctamine LTX and Plus reagent (Invitrogen), collected after 48 h in lysis buffer (20 mM Tris-HCl pH8.0, 137 mM NaCl, 1% NP-40, 2 mM EDTA, 1 μg/mL Leupeptin, 1 mM PMSF, 1 μM pepA, and 50 μM E-64), incubated with anti-c-Myc antibody (mouse IgG1κ, clone 9E10, 6 mg/mL, BioLegend) and protein G Sepharose beads (GE healthcare), immunoblotted with monoclonal anti-Myc (BioLegend, 1:1000) or polyclonal anti-DDDDK-tag (Rabbit IgG, 1:1000, MBL, PM020) antibodies, and detected by the SuperSignal West Pico Chemiluminescent Substrate (Pierce).

### Luciferase assays

HeLa cells (ATCC) were transfected using Lipofectamine LTX and Plus reagent with 400 ng reporter vector, 800 ng expression vectors, and 0.25 ng pSV-*Rluc* internal control vector^13^. Luciferase activity was measured 48 h after transient transfection according to the manufacturer’s instructions. Two duplicate, three independent experiments were performed.

### Promoter activity assays

HeLa cells were cotransfected with 400 ng of several *SEMA3C* promoter–pGL3 vectors and 0.25 ng pSV-*Rluc* internal control vector, together with 800 ng pcDNA3.1, Foxc1 or Foxc2 expression vector. The luciferase assay was performed as described previously^13^.

### Statistics

For luciferase assays, all experiments were performed at least in triplicate and data are reported as normalized relative light units (fold activation) together with the SEM. For promoter activity assays, all experiments were performed at least in triplicate and data are reported as the ratio of normalized relative light units for coexpression with Foxc1 or Foxc2 to that with mock (pcDNA3.1). An unpaired two-tailed t-test was used to calculate significant differences between two groups. If the data were normally distributed, a one-way ANOVA was used and multiple comparison correction analysis was performed using Tukey’s Post Hoc HSD test. A *p*-value of < 0.05 was considered statistically significant.

## Electronic supplementary material

Supplementary Figures and Legends

## References

[1] Gammill LS, Bronner-Fraser M (2003). Neural crest specification: migrating into genomics. Nature Reviews. Neuroscience.

[2] Kirby ML, Gale TF, Stewart DE (1983). Neural crest cells contribute to normal aorticopulmonary septation. Science.

[3] Kirby ML, Turnage KL, Hays BM (1985). Characterization of conotruncal malformations following ablation of “cardiac” neural crest. The Anatomical Record.

[4] Waldo KL (2001). Conotruncal myocardium arises from a secondary heart field. Development.

[5] Mjaatvedt CH (2001). The outflow tract of the heart is recruited from a novel heart-forming field. Developmental Biology.

[6] Kelly RG, Brown NA, Buckingham ME (2001). The arterial pole of the mouse heart forms from Fgf10-expressing cells in pharyngeal mesoderm. Developmental Cell.

[7] Kathiresan S, Srivastava D (2012). Genetics of human cardiovascular disease. Cell.

[8] Zhou Z (2017). Temporally Distinct Six2-Positive Second Heart Field Progenitors Regulate Mammalian Heart Development and Disease. Cell Reports.

[9] Yelbuz TM (2002). Shortened outflow tract leads to altered cardiac looping after neural crest ablation. Circulation.

[10] Tran TS, Kolodkin AL, Bharadwaj R (2007). Semaphorin regulation of cellular morphology. Annual Review of Cell and Developmental Biology.

[11] Feiner L (2001). Targeted disruption of semaphorin 3C leads to persistent truncus arteriosus and aortic arch interruption. Development.

[12] Brown CB (2001). PlexinA2 and semaphorin signaling during cardiac neural crest development. Development.

[13] Kodo K (2009). GATA6 mutations cause human cardiac outflow tract defects by disrupting semaphorin-plexin signaling. Proceedings of the National Academy of Sciences of the United States of America.

[14] Lepore JJ (2006). GATA-6 regulates semaphorin 3C and is required in cardiac neural crest for cardiovascular morphogenesis. The Journal of Clinical Investigation.

[15] Kothary R (1988). A transgene containing lacZ inserted into the dystonia locus is expressed in neural tube. Nature.

[16] Yamagishi H (2003). Tbx1 is regulated by tissue-specific forkhead proteins through a common Sonic hedgehog-responsive enhancer. Genes & Development.

[17] Seo S, Kume T (2006). Forkhead transcription factors, Foxc1 and Foxc2, are required for the morphogenesis of the cardiac outflow tract. Developmental Biology.

[18] Maeda J, Yamagishi H, McAnally J, Yamagishi C, Srivastava D (2006). Tbx1 is regulated by forkhead proteins in the secondary heart field. Developmental Dynamics.

[19] Chapman DL (1996). Expression of the T-box family genes, Tbx1-Tbx5, during early mouse development. Developmental Dynamics.

[20] Garg V (2001). Tbx1, a DiGeorge syndrome candidate gene, is regulated by sonic hedgehog during pharyngeal arch development. Developmental Biology.

[21] Yamagishi H, Srivastava D (2003). Unraveling the genetic and developmental mysteries of 22q11 deletion syndrome. Trends in Molecular Medicine.

[22] Jerome LA, Papaioannou VE (2001). DiGeorge syndrome phenotype in mice mutant for the T-box gene, Tbx1. Nature Genetics.

[23] Lindsay EA (2001). Tbx1 haploinsufficieny in the DiGeorge syndrome region causes aortic arch defects in mice. Nature.

[24] Merscher S (2001). TBX1 is responsible for cardiovascular defects in velo-cardio-facial/DiGeorge syndrome. Cell.

[25] Theveniau-Ruissy M (2008). The del22q11.2 candidate gene Tbx1 controls regional outflow tract identity and coronary artery patterning. Circulation Research.

[26] Hu T (2004). Tbx1 regulates fibroblast growth factors in the anterior heart field through a reinforcing autoregulatory loop involving forkhead transcription factors. Development.

[27] Toyofuku T (2008). Repulsive and attractive semaphorins cooperate to direct the navigation of cardiac neural crest cells. Developmental Biology.

[28] Abu-Issa R, Smyth G, Smoak I, Yamamura K, Meyers EN (2002). Fgf8 is required for pharyngeal arch and cardiovascular development in the mouse. Development.

[29] Frank DU (2002). An Fgf8 mouse mutant phenocopies human 22q11 deletion syndrome. Development.

[30] Park EJ (2006). Required, tissue-specific roles for Fgf8 in outflow tract formation and remodeling. Development.

[31] Ilagan R (2006). Fgf8 is required for anterior heart field development. Development.

[32] Sato A (2011). FGF8 signaling is chemotactic for cardiac neural crest cells. Developmental Biology.

[33] Suzuki-Hirano A, Harada H, Sato T, Nakamura H (2010). Activation of Ras-ERK pathway by Fgf8 and its downregulation by Sprouty2 for the isthmus organizing activity. Developmental Biology.

[34] Kawakami Y (2003). MKP3 mediates the cellular response to FGF8 signalling in the vertebrate limb. Nature Cell Biology.

[35] Danielian PS, Muccino D, Rowitch DH, Michael SK, McMahon AP (1998). Modification of gene activity in mouse embryos in utero by a tamoxifen-inducible form of Cre recombinase. Current Biology.

[36] Kawamoto S (2000). A novel reporter mouse strain that expresses enhanced green fluorescent protein upon Cre-mediated recombination. FEBS Letters.

[37] Kume T (1998). The forkhead/winged helix gene Mf1 is disrupted in the pleiotropic mouse mutation congenital hydrocephalus. Cell.

[38] Kirby ML, Hutson MR (2010). Factors controlling cardiac neural crest cell migration. Cell Adhesion & Migration.

[39] Plein A (2015). Neural crest-derived SEMA3C activates endothelial NRP1 for cardiac outflow tract septation. The Journal of Clinical Investigation.

[40] Brown CB (2004). Cre-mediated excision of Fgf8 in the Tbx1 expression domain reveals a critical role for Fgf8 in cardiovascular development in the mouse. Developmental Biology.

[41] Kelly ML, Astsaturov A, Rhodes J, Chernoff J (2014). A Pak1/Erk signaling module acts through Gata6 to regulate cardiovascular development in zebrafish. Developmental Cell.

[42] Kothary R (1989). Inducible expression of an hsp68-lacZ hybrid gene in transgenic mice. Development.

[43] Hamburger V, Hamilton HL (1951). A series of normal stages in the development of the chick embryo. Journal of Morphology.

[44] Miyagawa-Tomita S, Waldo K, Tomita H, Kirby ML (1991). Temporospatial study of the migration and distribution of cardiac neural crest in quail-chick chimeras. The American Journal of Anatomy.

[45] Waldo K, Miyagawa-Tomita S, Kumiski D, Kirby ML (1998). Cardiac neural crest cells provide new insight into septation of the cardiac outflow tract: aortic sac to ventricular septal closure. Developmental Biology.

[46] Nagoshi N (2008). Ontogeny and multipotency of neural crest-derived stem cells in mouse bone marrow, dorsal root ganglia, and whisker pad. Cell Stem Cell.

[47] Pfaltzgraff ER, Mundell NA, Labosky PA (2012). Isolation and culture of neural crest cells from embryonic murine neural tube. Journal of Visualized Experiments.

[48] Lizee G (2003). Real-time quantitative reverse transcriptase-polymerase chain reaction as a method for determining lentiviral vector titers and measuring transgene expression. Human Gene Therapy.

[49] Monzen K (2001). Smads, TAK1, and their common target ATF-2 play a critical role in cardiomyocyte differentiation. The Journal of Cell Biology.

