# Supplementary material for: Regulation of Sema3c and the Interaction between Cardiac Neural Crest and Second Heart Field during Outflow Tract Development
Source: Sci Rep. 2017 Jul 28;7:6771. doi: 10.1038/s41598-017-06964-9 (PMC5533775; doi:10.1038/s41598-017-06964-9)
Supplement: Supplementary file 1 — Supplementary Figures and Legends [file 41598_2017_6964_MOESM1_ESM.pdf]

SUPPLEMENTARY INFORMATION

Regulation of Sema3c and the Interaction between Cardiac Neural Crest and Second Heart Field during Outflow Tract Development

Kazuki Kodo[1*], Shinsuke Shibata[2], Sachiko Miyagawa-Tomita[3], Sang-Ging Ong[4], Hiroshi Takahashi[5], Tsutomu Kume[6], Hideyuki Okano[2], Rumiko Matsuoka[3] & Hiroyuki Yamagishi[1*]

[1]Department of Pediatrics, Division of Pediatric Cardiology and [2]Department of Physiology, Keio University School of Medicine, Tokyo 160-8582, Japan

[3]Department of Pediatric Cardiology, Tokyo Women's Medical University, Tokyo 162-8666, Japan

[4]Stanford Cardiovascular Institute, Stanford University School of Medicine, Stanford, CA 94305-5111, USA

[5]Department of Neurology, National Hospital Organization, Tottori Medical Center, Tottori, Tottori 689-0203, Japan

[6]Feinberg Cardiovascular Research Institute, Feinberg School of Medicine, Northwestern University, Chicago, IL 60611, USA

[*]corresponding. kazukikodo.a3@keio.jp or hyamag@keio.jp

Supplementary Figure Legends

Supplementary Figure S1. Foxc1 and Foxc2 positively regulate *Sema3c* promoter/enhancer. Luciferase expression under control of the *Sema3c* genomic DNA fragments (from -711 to +68). Foxc1 and Foxc2 exhibited significant increase in transcription activity compared with the control (pcDNA) (Foxc1, p=0.0073; Foxc2, p=0.0030; 2-tailed unpaired t-test). Tbx5, Nkx2.5 and Srf also showed transcriptional activation of *Sema3c* promoter although these activations were weaker than Foxc1/Foxc2 (Tbx5, p=0.00034; Nkx2.5, p=0.021; Srf, p=0.0066; 2-tailed unpaired t-test). Two duplicate, three independent studies were performed. Error bars represent s.e.m.

Supplementary Figure S2. Tbx1 synergistically activates the *Sema3c* 5' outflow tract enhancer with Foxc1 and Foxc2. **(A)** Luciferase expression under the control of the *Sema3c* genomic DNA fragments (from -711 to +68). Tbx1 itself showed no significant activation on Sema3c outflow tract enhancer. Co-overexpression of Tbx1 with Foxc1 and Foxc2 exhibited significant increase in transcriptional activity. Six independent studies were performed. ***$p < 0.005$. Error bars represent s.e.m. **(B)** Immunoprecipitation (IP) with Myc-tagged Tbx1 revealed an association with Flag-tagged Foxc1 (left), whereas no direct binding between Tbx1 and Foxc2 was observed (right). IgG, immunoglobulin band.

Supplementary Figure S3. Aberrant mRNA expression of *Sema3c* in pharyngeal arch region in the hypomorphic state of *Tbx1* (*Tbx1*$^{neo/neo}$). DIG-labeled whole mount

in situ hybridization of Sema3c mRNA in *Tbx1*$^{neo/+}$ (upper, left) and *Tbx1*$^{neo/neo}$ (lower, left) mouse embryos at E10.5. Higher magnification bilateral views of pharyngeal arch region (white box) are shown in right lane. Scale bars, 500μm.

Supplementary Figure S4. Characterization of enhancer for expression in pharyngeal arch region using Sema3c3' flanking fragment. **(A)** *Sema3c* 3'-lacZ transgene construct (*Hsp68* basal promoter and *lacZ* gene followed by 3' region from +155988 to +157878). **(B)** The summary of the number of F0 transgenic embryos with the specific lacZ expression in the out flow tract (Oft) and pharyngeal arch (PAA) region with the total number of F0 lacZ positive embryos. **(C)** Left lateral views of the pharyngeal arch region of representative Sema3c 3'-lacZ transgenic embryos. Scale bars, 500μm.

Supplementary Figure S5. Abnormal aggregation by Sema3c overexpression in cNCCs. Representative images of pre- and post-lentiviral transduction cNCC explants. Explants were infected with control or Sema3c overexpression lentivirus. 24 hrs after lentivirus transduction, Sema3c-overexpressed cNCC explants (Sema3c-OE) showed aggregation (black arrowheads) whereas Sema3c neutralizing antibody prevented the aggregation.

Supplementary Figure S6. Fgf8 shows no significant effect on the differentiation in the thoracic pharyngeal arches-derived neural spheres. **(A)** Expression pattern of EGFP in Wnt1-Cre/floxed-EGFP mice at E10.5 and derived neural spheres after 7 days of culture in sphere-forming media (cut-in). The area encircled by white lines indicates the dissected pharyngeal arch region. Scale bars, 100 mm. **(B)** EGFP-positive spheres maintained

their abilities to differentiate into neurons (green), glial cells (red) and myofibroblasts (blue). Scale bars, 100 mm. **(C)** Differentiation potential of clonal spheres. Spheres showed no significant tendency towards the formation of glia, neuron or smooth muscle-lineage differentiation with or without addition of Fgf8. Five independent studies were performed and a total of 182 spheres for control and 186 spheres for Fgf8 group were validated. ns, no significant difference. Statistical analyses were conducted using unpaired two-tailed *t*-test. Error bars represent s.e.m.

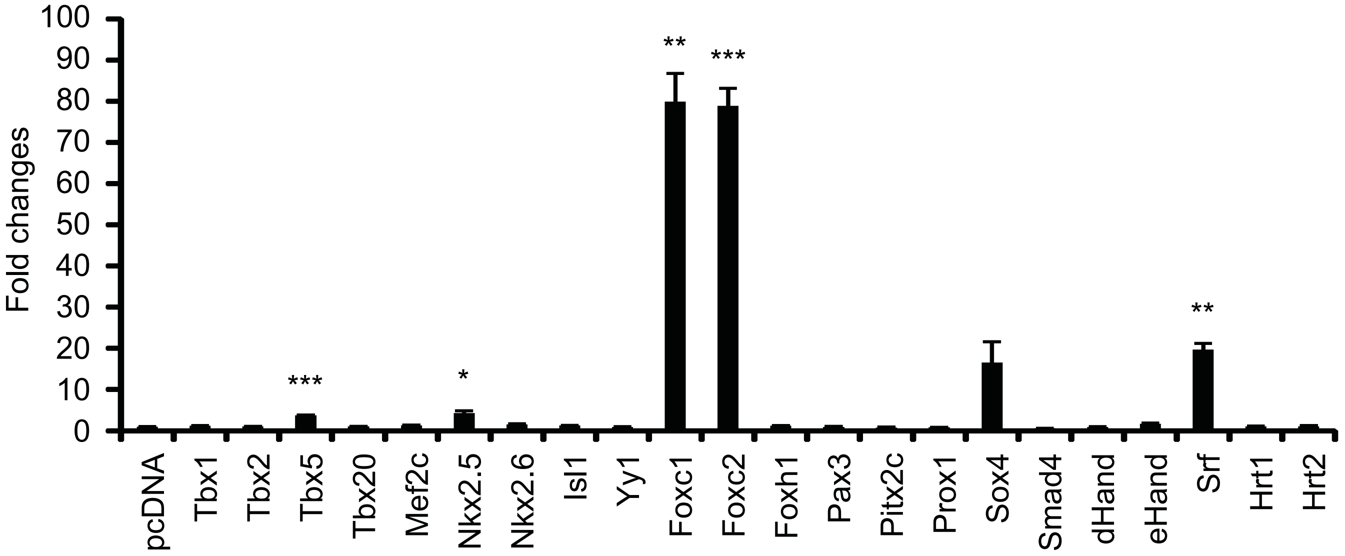

Supplementary Figure S1

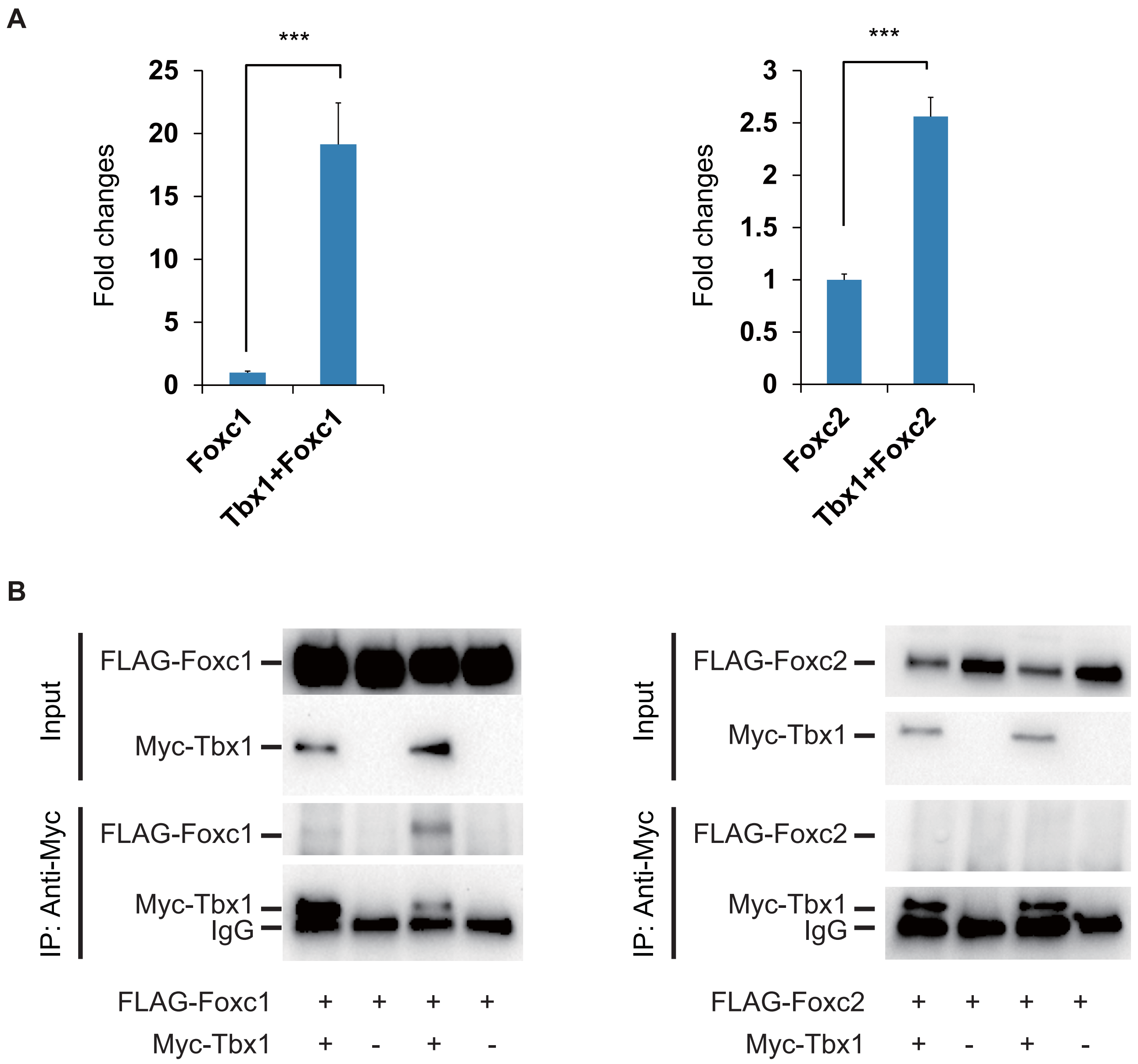

Supplementary Figure S2

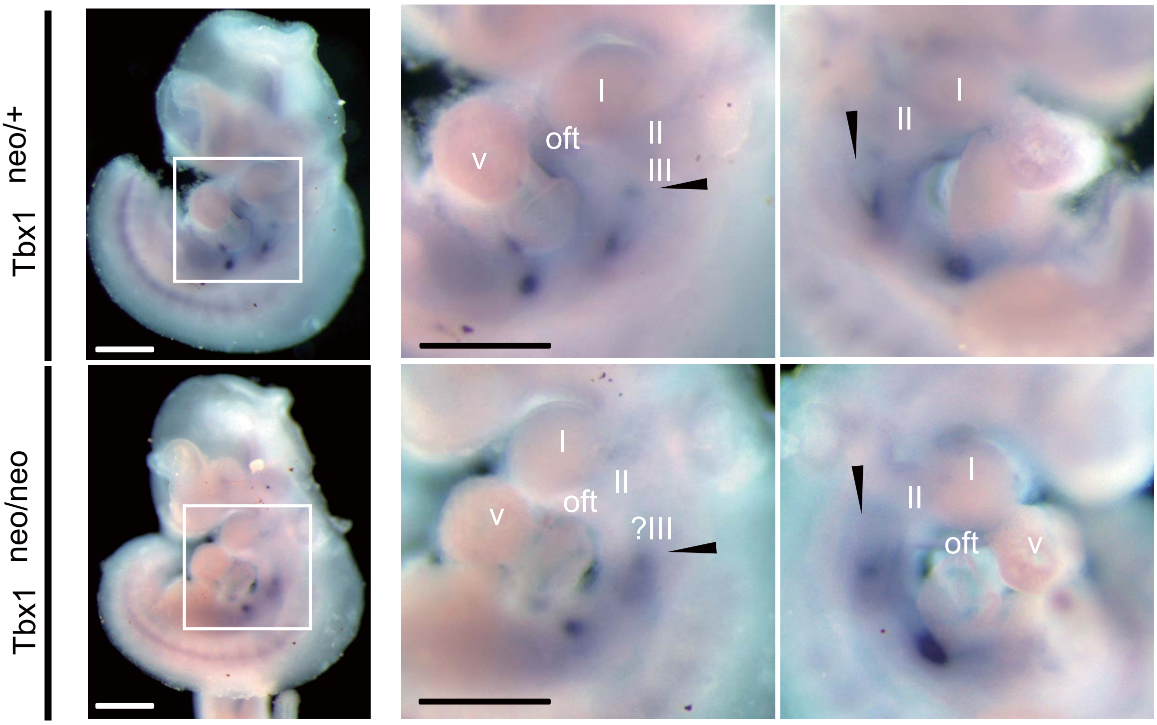

Supplementary Figure S3

A

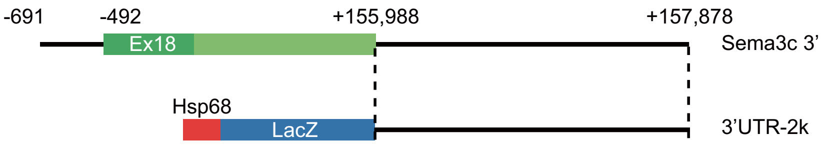

B

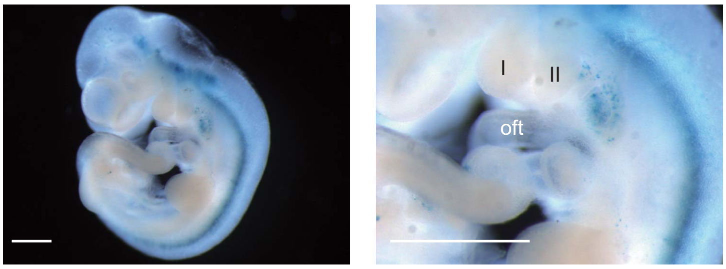

C

	# of embryos with lacZ expression in Oft region	# of embryos with lacZ expression in pharyngeal arch region	Total # of lacZ positive embryos
3'UTR-2k	1	5	6

Supplementary Figure S4

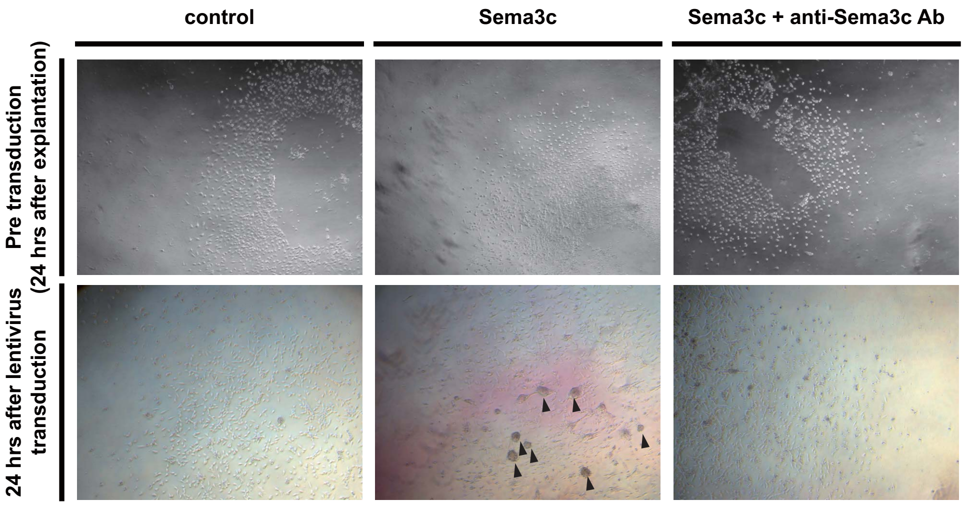

Supplementary Figure S5

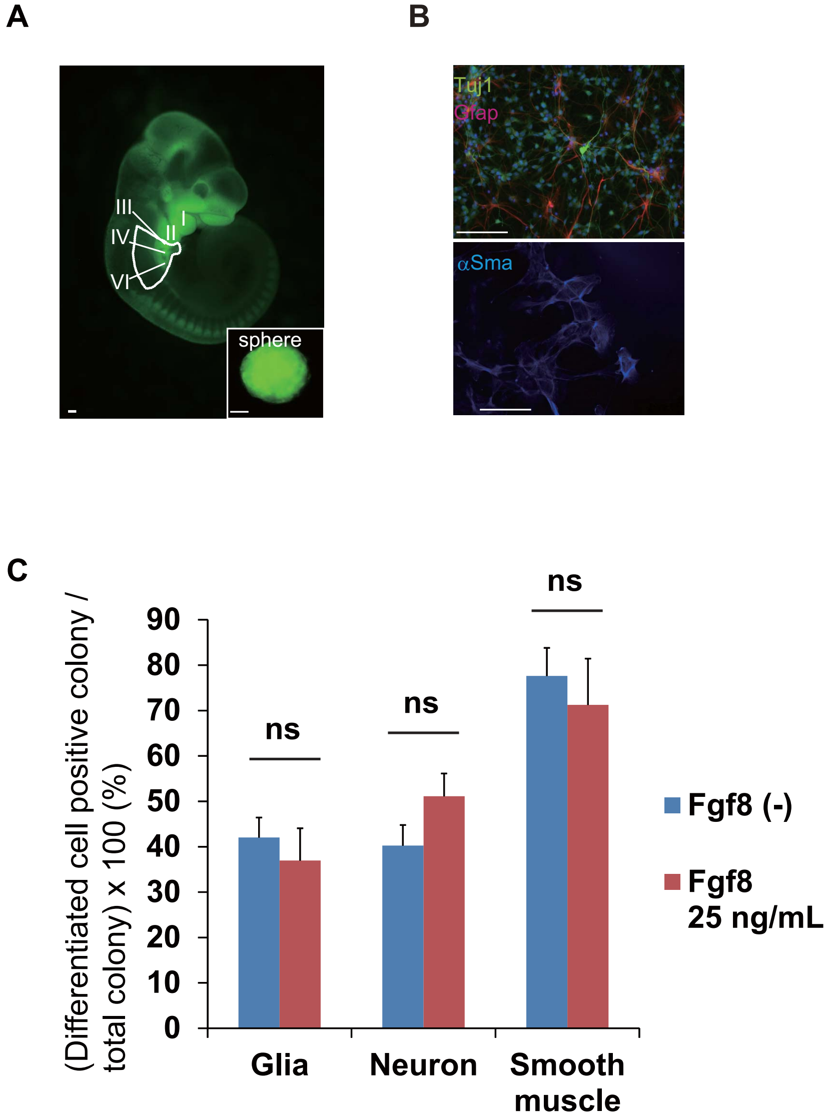

Supplementary Figure S6
